# Supplementary material for: Analysis of metagenome-assembled viral genomes from the human gut reveals diverse putative CrAss-like phages with unique genomic features
Source: Nat Commun. 2021 Feb 16;12:1044. doi: 10.1038/s41467-021-21350-w (PMC7886860; doi:10.1038/s41467-021-21350-w)

Supplementary figures for:

Analysis of metagenome-assembled viral genomes from the human gut reveals diverse putative CrAss-like phages with unique genomic features

Natalya Yutin[1], Sean Benler[1], Sergei A. Shmakov[1], Yuri I. Wolf[1], Igor Tolstoy[1], Mike Rayko[2], Dmitry Antipov[2], Pavel A. Pevzner[3], Eugene V. Koonin[1*]

[1], National Center for Biotechnology Information, National Library of Medicine, Bethesda, Maryland 20894,USA;
[2], Center for Algorithmic Biotechnology, Institute for Translational Biomedicine, St. Petersburg State University, 199004 St. Petersburg, Russia ;
[3], Department of Computer Science and Engineering, University of California-San Diego, La Jolla, CA 92093, USA.

*Correspondence : koonin@ncbi.nlm.nih.gov

Supplementary Figure 1

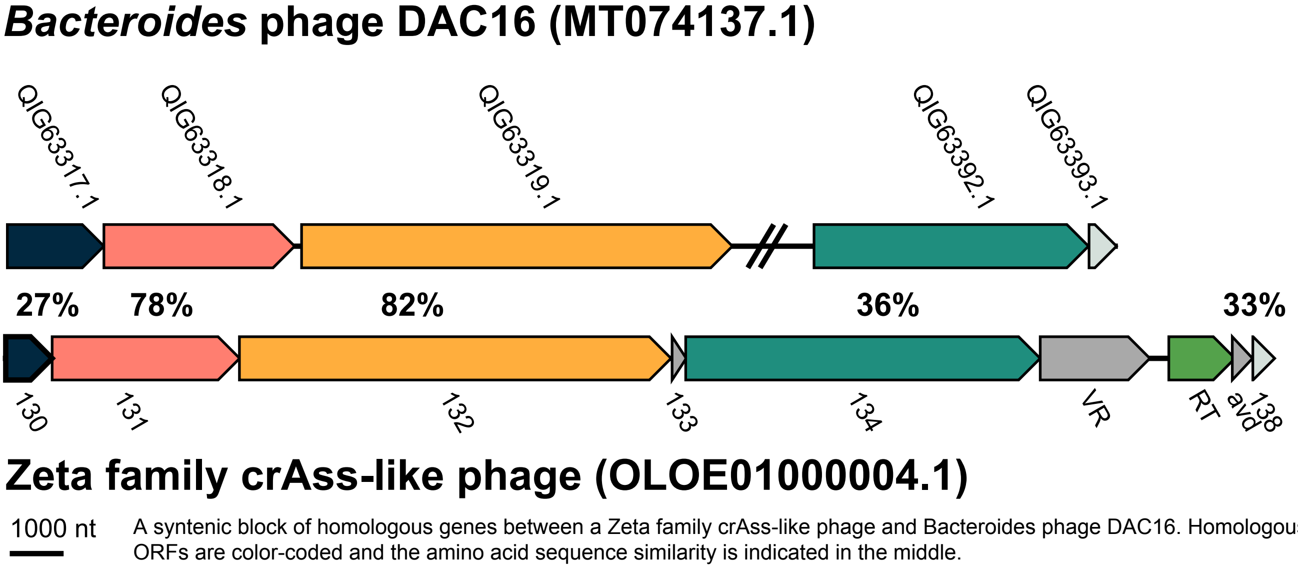

A syntenic block of homologous genes between a Zeta family crAss-like phage and Bacteroides phage DAC16. Homologous ORFs are color-coded and the amino acid sequence similarity is indicated in the middle.

Supplementary Figure 2

A

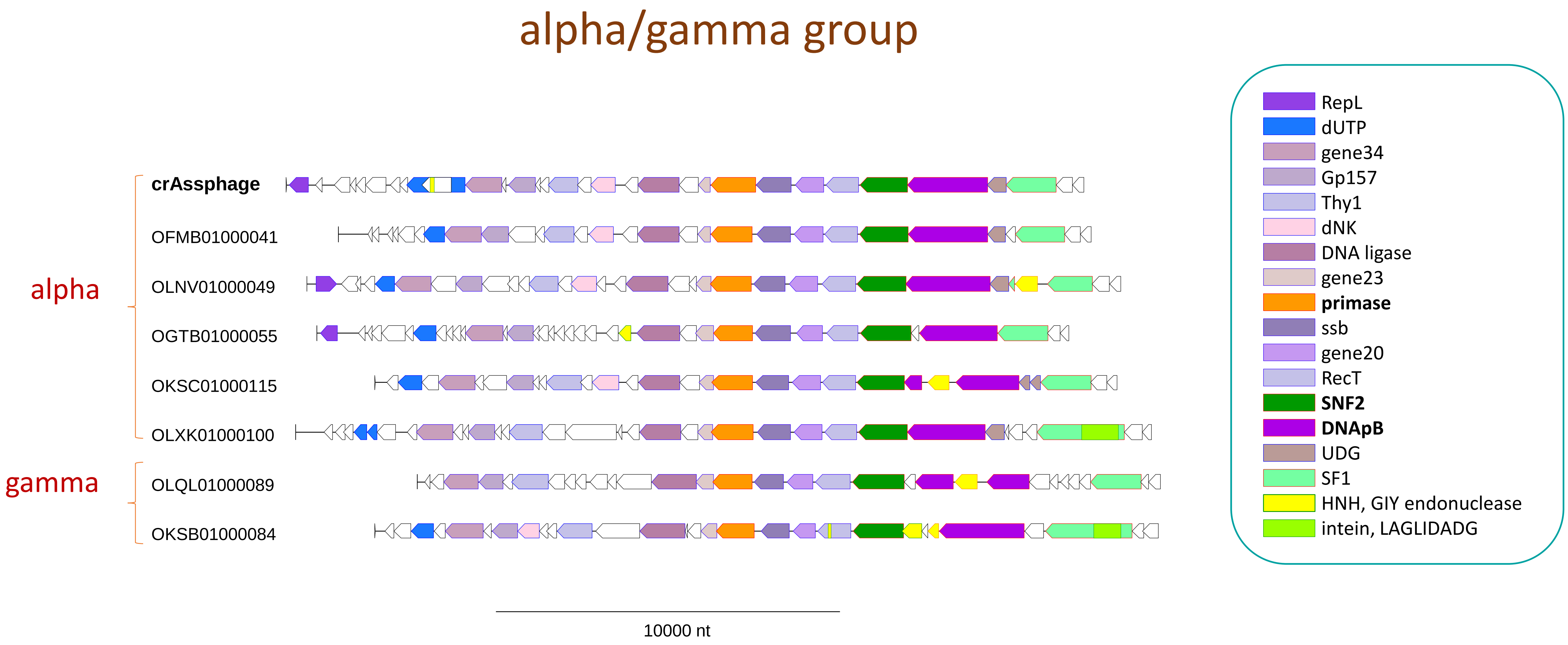

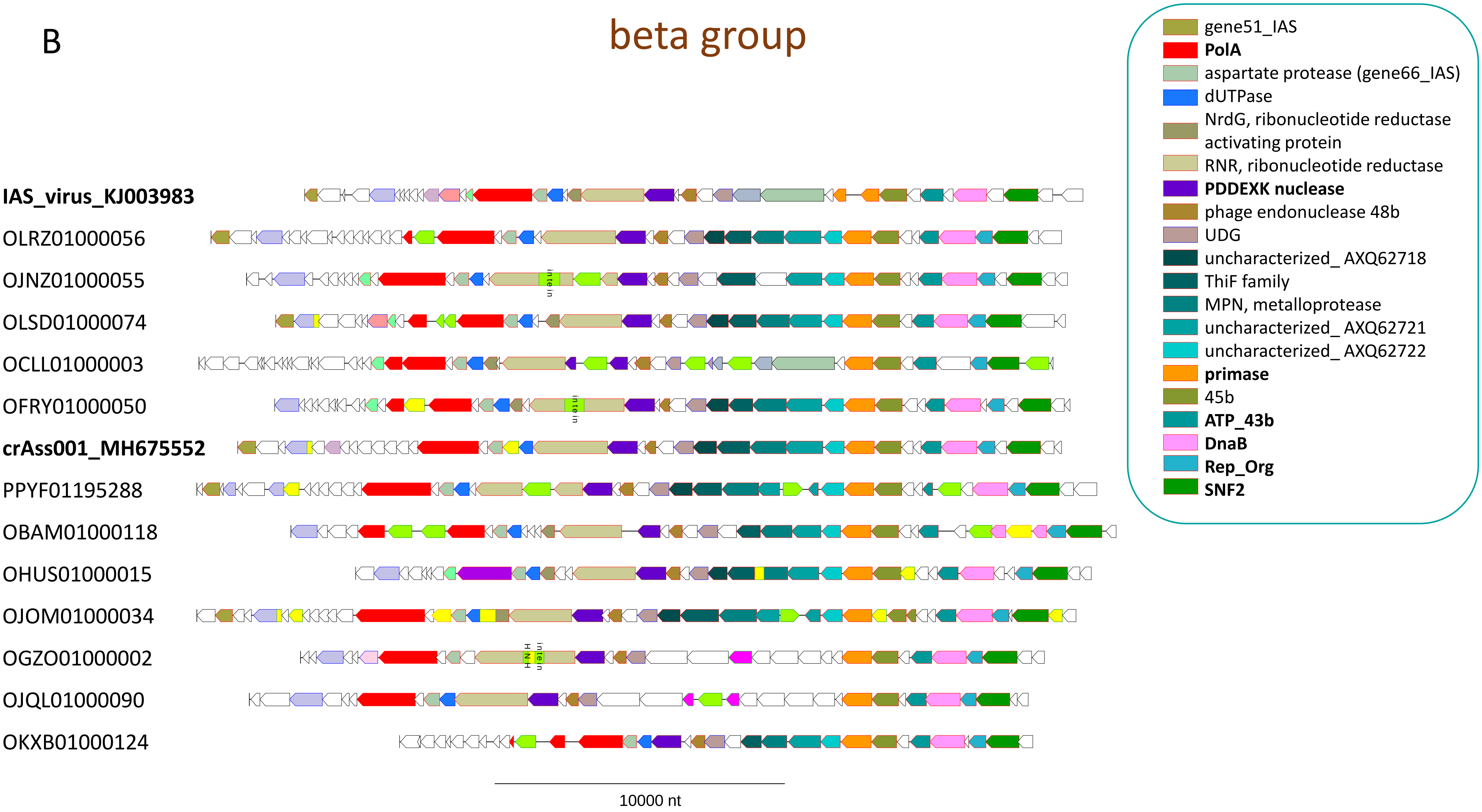
B
beta group
IAS_virus_KJ003983
OLRZ01000056
OJNZ01000055
OLSD01000074
OCLL01000003
OFRY01000050
crAss001_MH675552
PPYF01195288
OBAM01000118
OHUS01000015
OJOM01000034
OGZO01000002
OJQL01000090
OKXB01000124
intein
HNH
10000 nt
gene51_IAS
PolA
aspartate protease (gene66_IAS)
dUTPase
NrdG, ribonucleotide reductase activating protein
RNR, ribonucleotide reductase
PDDEXK nuclease
phage endonuclease 48b
UDG
uncharacterized_ AXQ62718
ThiF family
MPN, metalloprotease
uncharacterized_ AXQ62721
uncharacterized_ AXQ62722
primase
45b
ATP_43b
DnaB
Rep_Org
SNF2

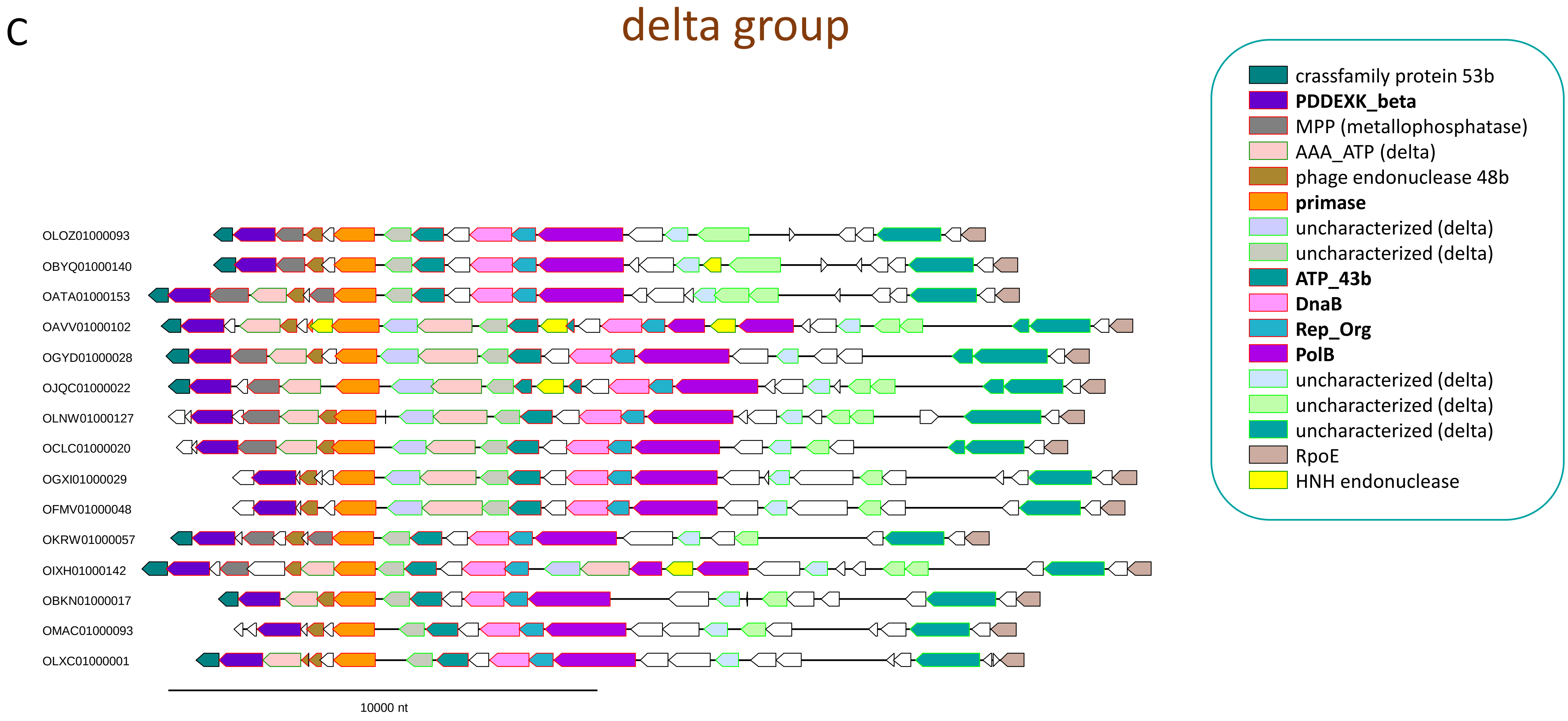
c
delta group
OLOZ01000093
OBYQ01000140
OATA01000153
OAVV01000102
OGYD01000028
OJQC01000022
OLNW01000127
OCLC01000020
OGXI01000029
OFMV01000048
OKRW01000057
OIXH01000142
OBKN01000017
OMAC01000093
OLXC01000001
10000 nt
crassfamily protein 53b
PDDEXK_beta
MPP (metallophosphatase)
AAA_ATP (delta)
phage endonuclease 48b
primase
uncharacterized (delta)
uncharacterized (delta)
ATP_43b
DnaB
Rep_Org
PolB
uncharacterized (delta)
uncharacterized (delta)
uncharacterized (delta)
RpoE
HNH endonuclease

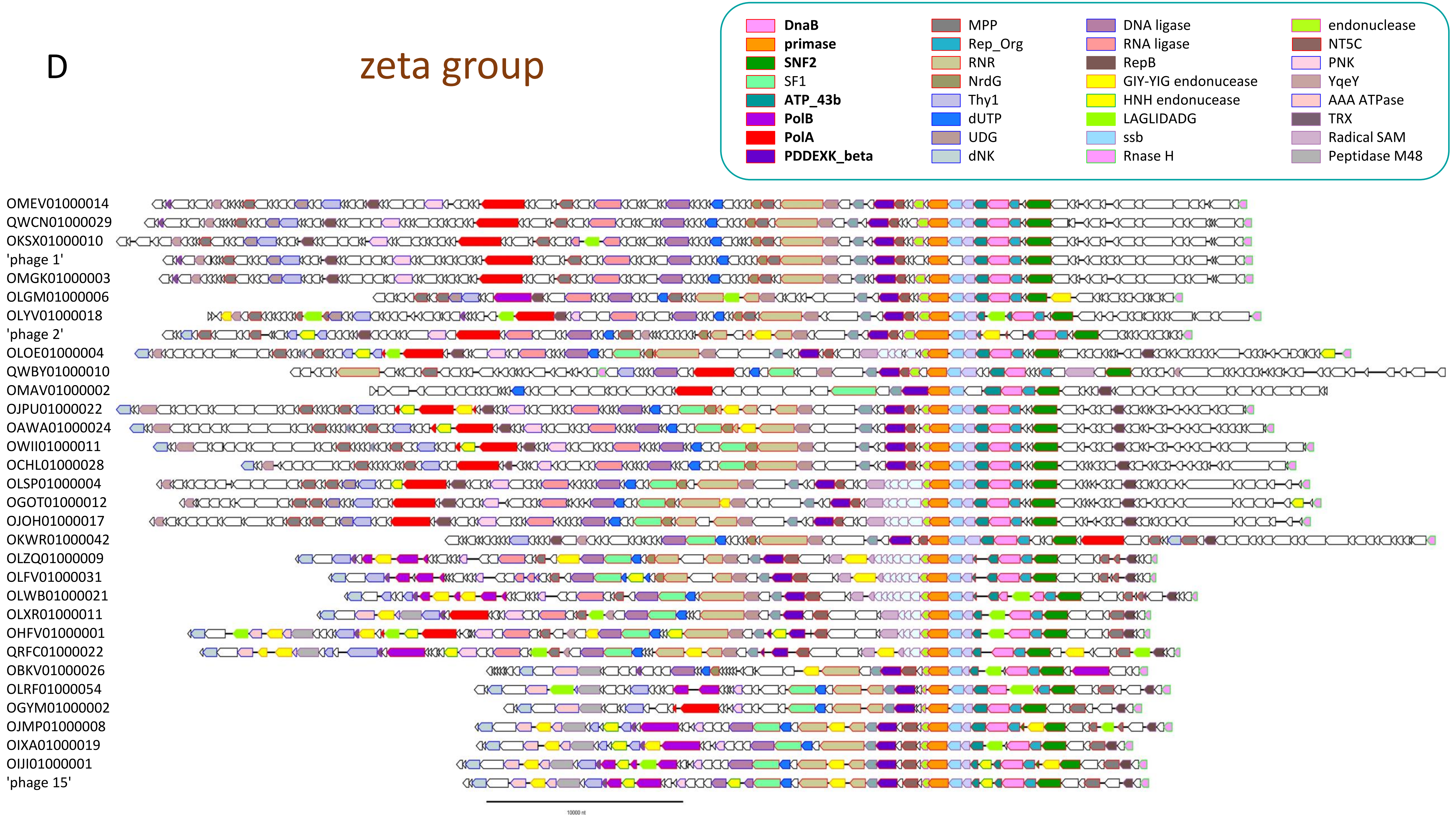
D
zeta group
DnaB
primase
SNF2
SF1
ATP_43b
PolB
PolA
PDDEXK_beta
MPP
Rep_Org
RNR
NrdG
Thy1
dUTP
UDG
dNK
DNA ligase
RNA ligase
RepB
GIY-YIG endonucease
HNH endonucease
LAGLIDADG
ssb
Rnase H
endonuclease
NT5C
PNK
YqeY
AAA ATPase
TRX
Radical SAM
Peptidase M48
OMEV01000014
QWCN01000029
OKSX01000010
'phage 1'
OMGK01000003
OLGM01000006
OLYV01000018
'phage 2'
OLOE01000004
QWBY01000010
OMAV01000002
OJPU01000022
OAWA01000024
OWII01000011
OCHL01000028
OLSP01000004
OGOT01000012
OJOH01000017
OKWR01000042
OLZQ01000009
OLFV01000031
OLWB01000021
OLXR01000011
OHFV01000001
QRFC01000022
OBKV01000026
OLRF01000054
OGYM01000002
OJMP01000008
OIXA01000019
OIJI01000001
'phage 15'
10000 nt

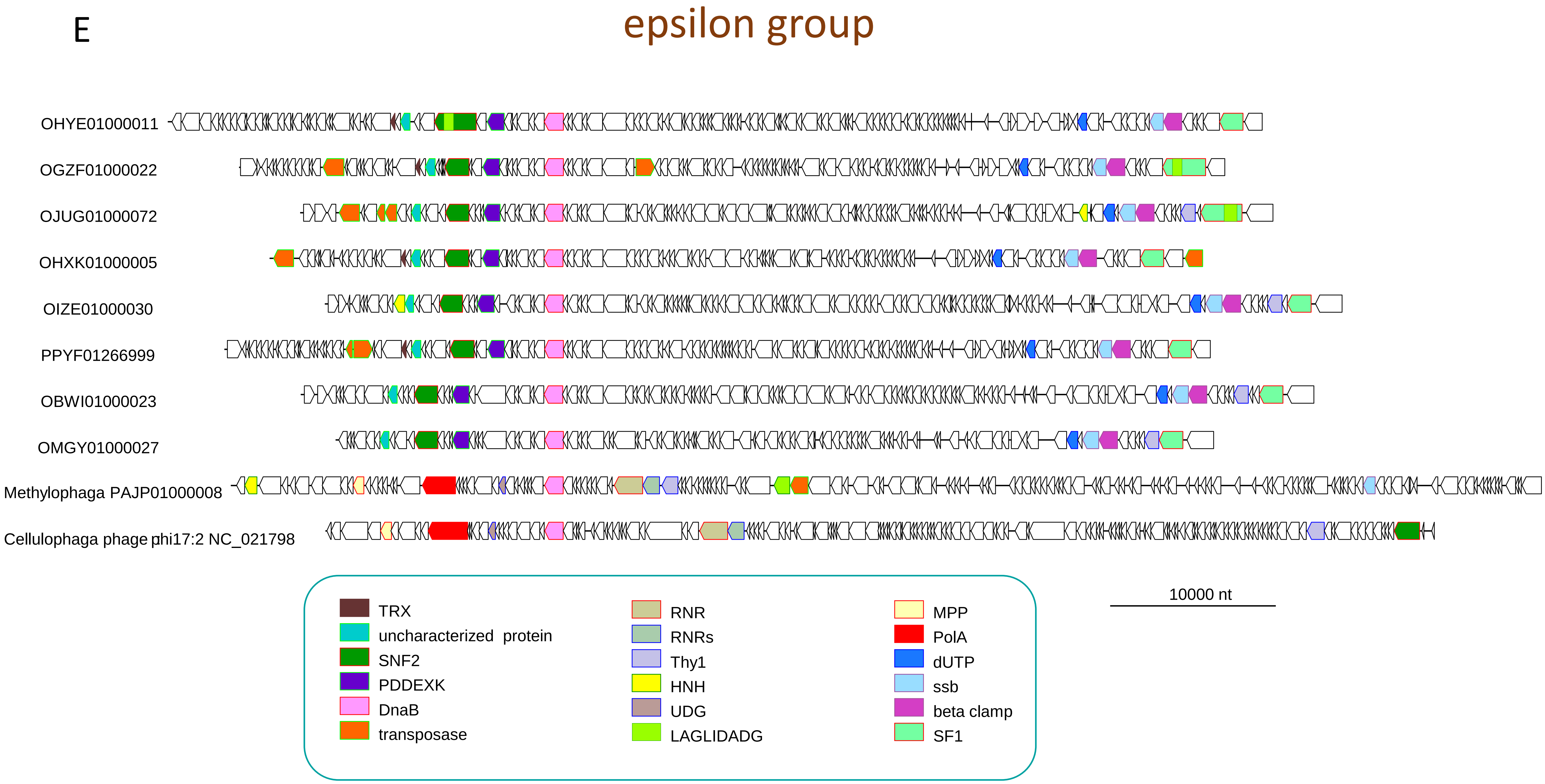

Group-specific genome maps of the replication gene block of the crAss-like phages. A, Alpha and Gamma groups; B, Beta group; C, Delta group; D, Zeta group; E, Epsilon group

Supplementary Figure 3

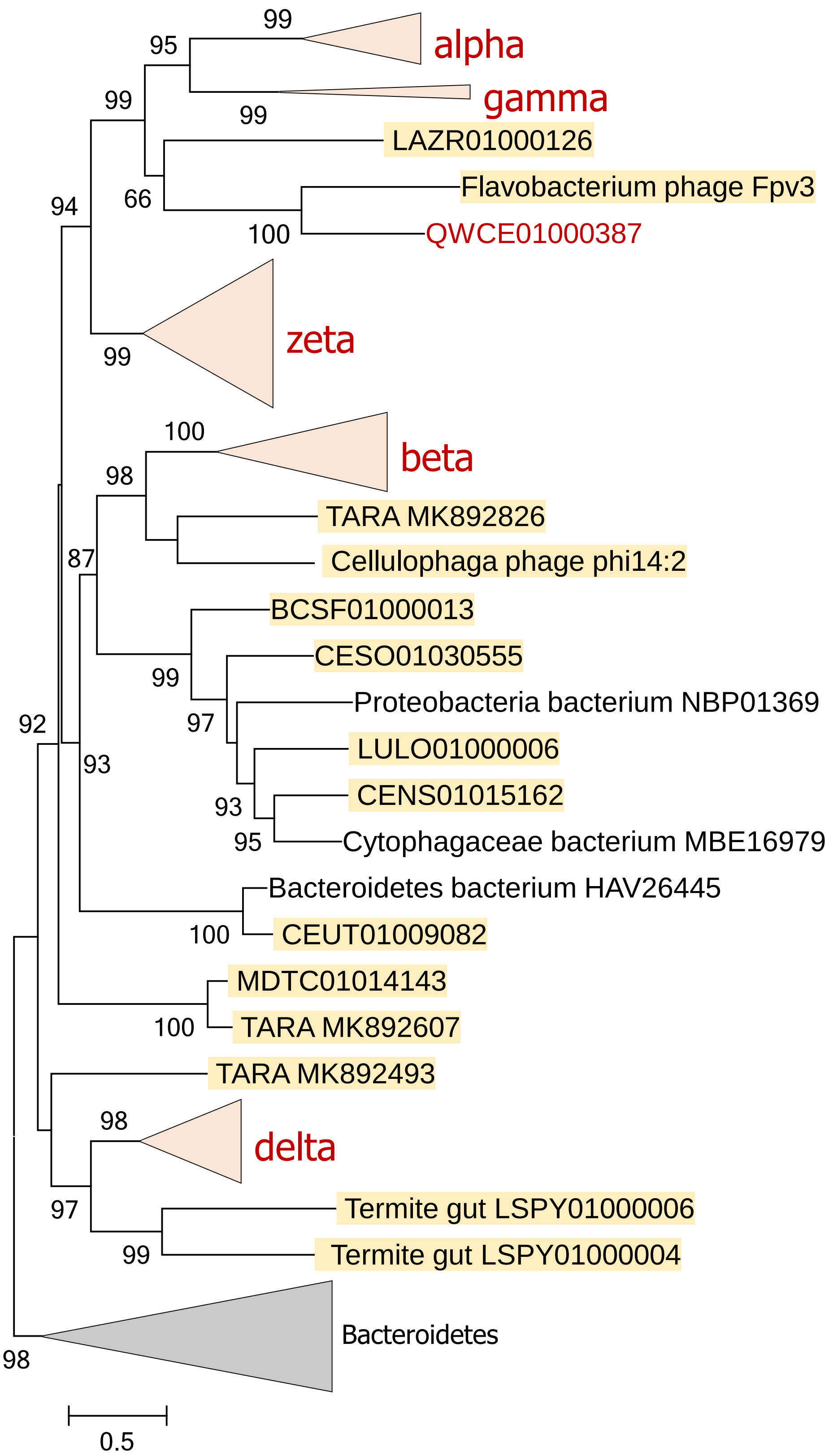

Phylogenetic tree of the DnaG family primase.
Previously analyzed crAss-like sequences are highlighted in yellow.

Supplementary Figure 4

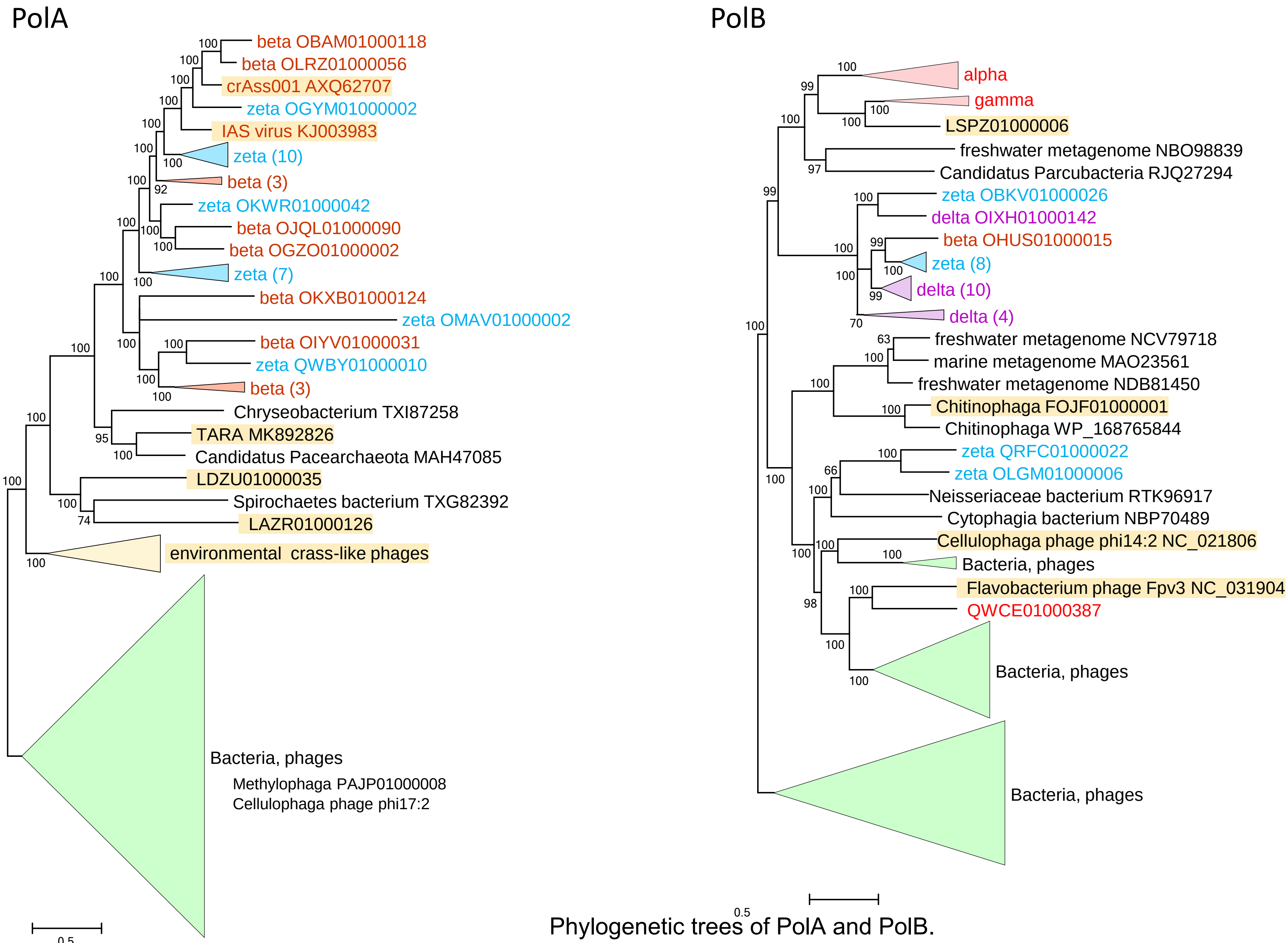

Phylogenetic trees of PolA and PolB.
Previously analyzed crAss-like sequences are highlighted in

Supplementary Figure 5

Two examples of in situ DNAP replacement in crAss-like phages.
A, Genomic organization around the DNAP genes
B, Nucleotide sequence alignments of the 5'-terminal regions of DNAP genes and the upstream regions

A

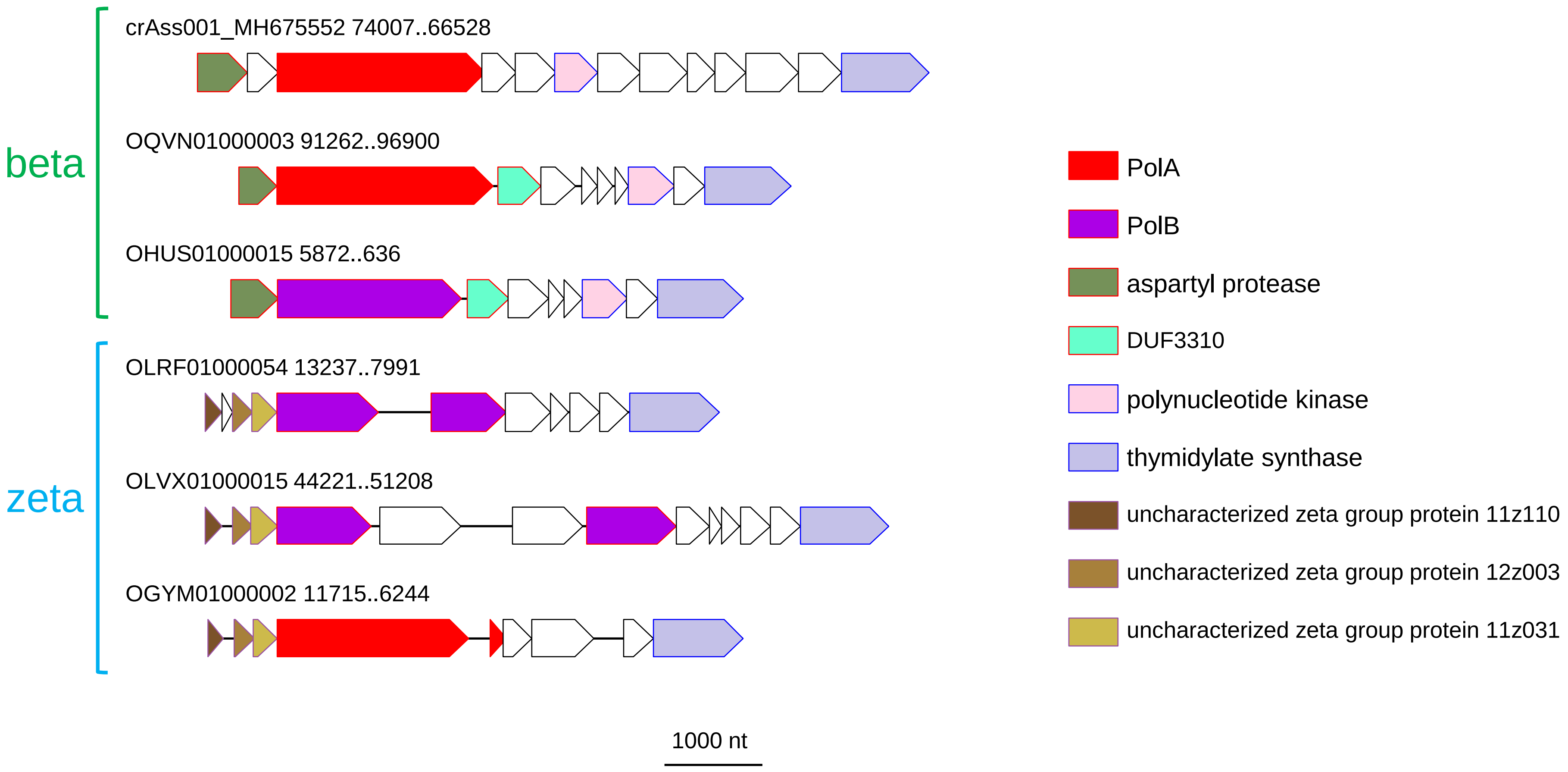

B

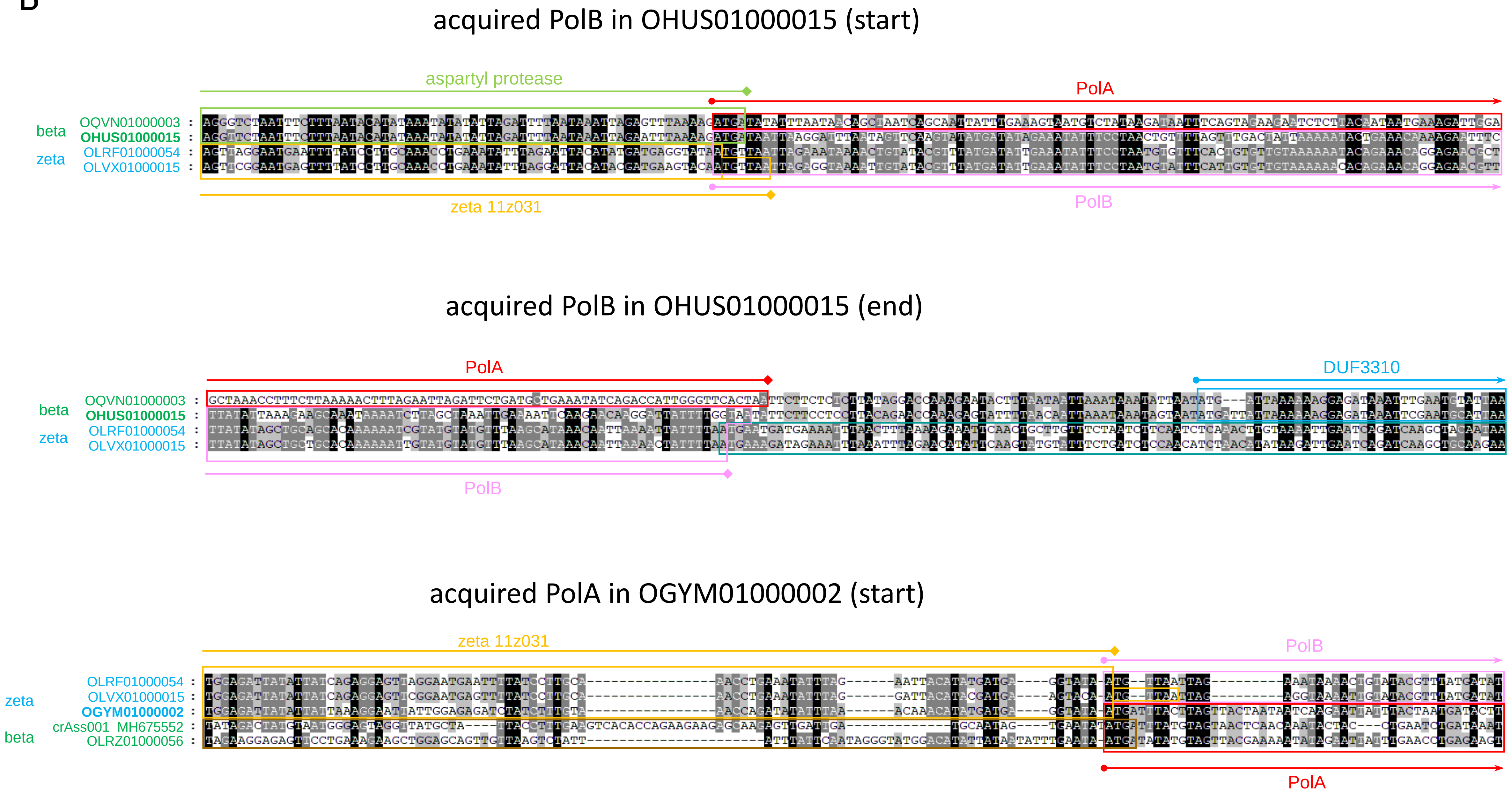
acquired PolB in OHUS01000015 (start)
aspartyl protease
PolA
beta
OQVN01000003
OHUS01000015
zeta
OLRF01000054
OLVX01000015
zeta 11z031
PolB
acquired PolB in OHUS01000015 (end)
PolA
DUF3310
beta
OQVN01000003
OHUS01000015
zeta
OLRF01000054
OLVX01000015
PolB
acquired PolA in OGYM01000002 (start)
zeta 11z031
PolB
zeta
OLRF01000054
OLVX01000015
OGYM01000002
crAss001 MH675552
beta
OLRZ01000056
PolA

Supplementary Figure 6

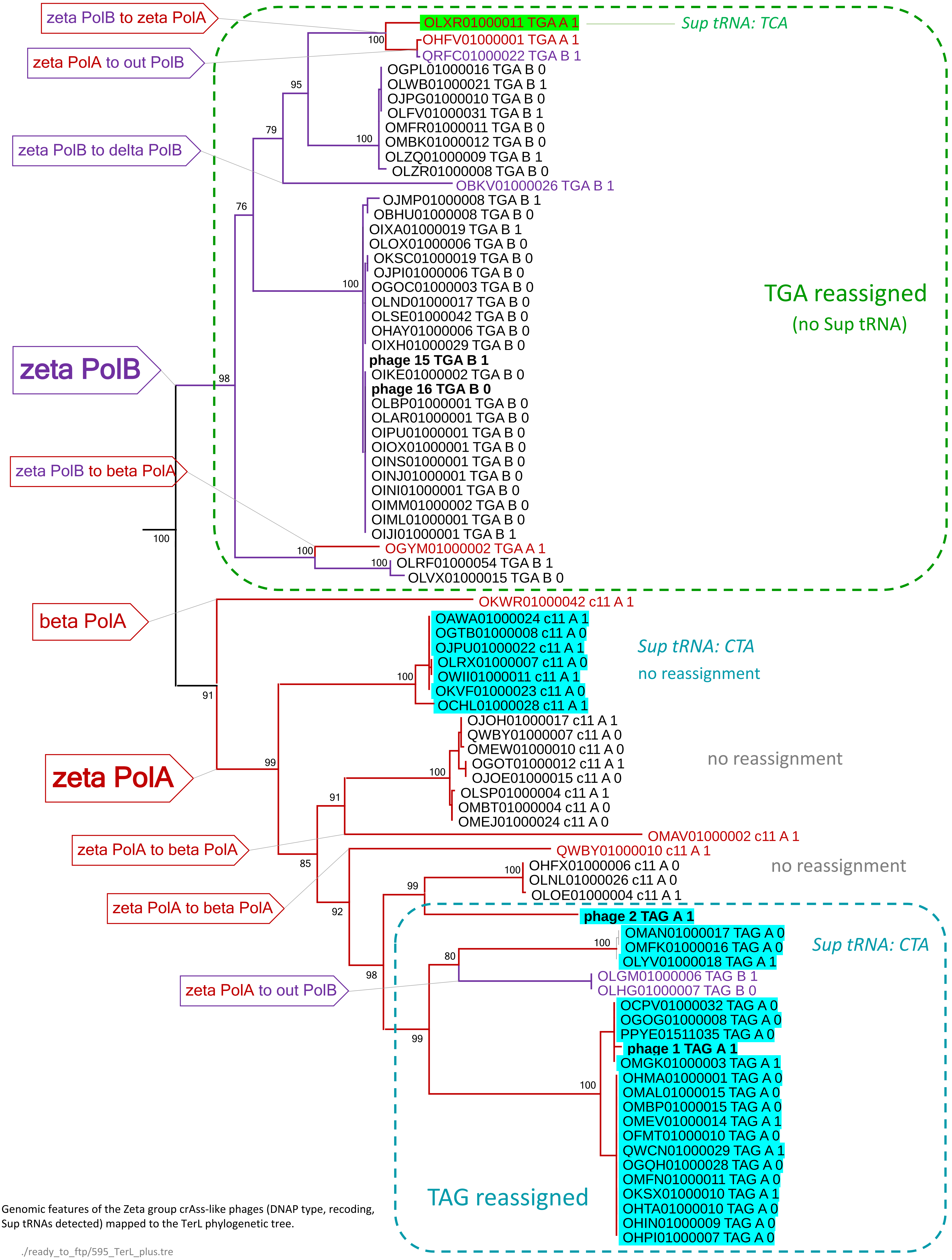

Genomic features of the Zeta group crAss-like phages (DNAP type, recoding, Sup tRNAs detected) mapped to the TerL phylogenetic tree.

./ready_to_ftp/595_TerL_plus.tre

Supplementary Figure 7

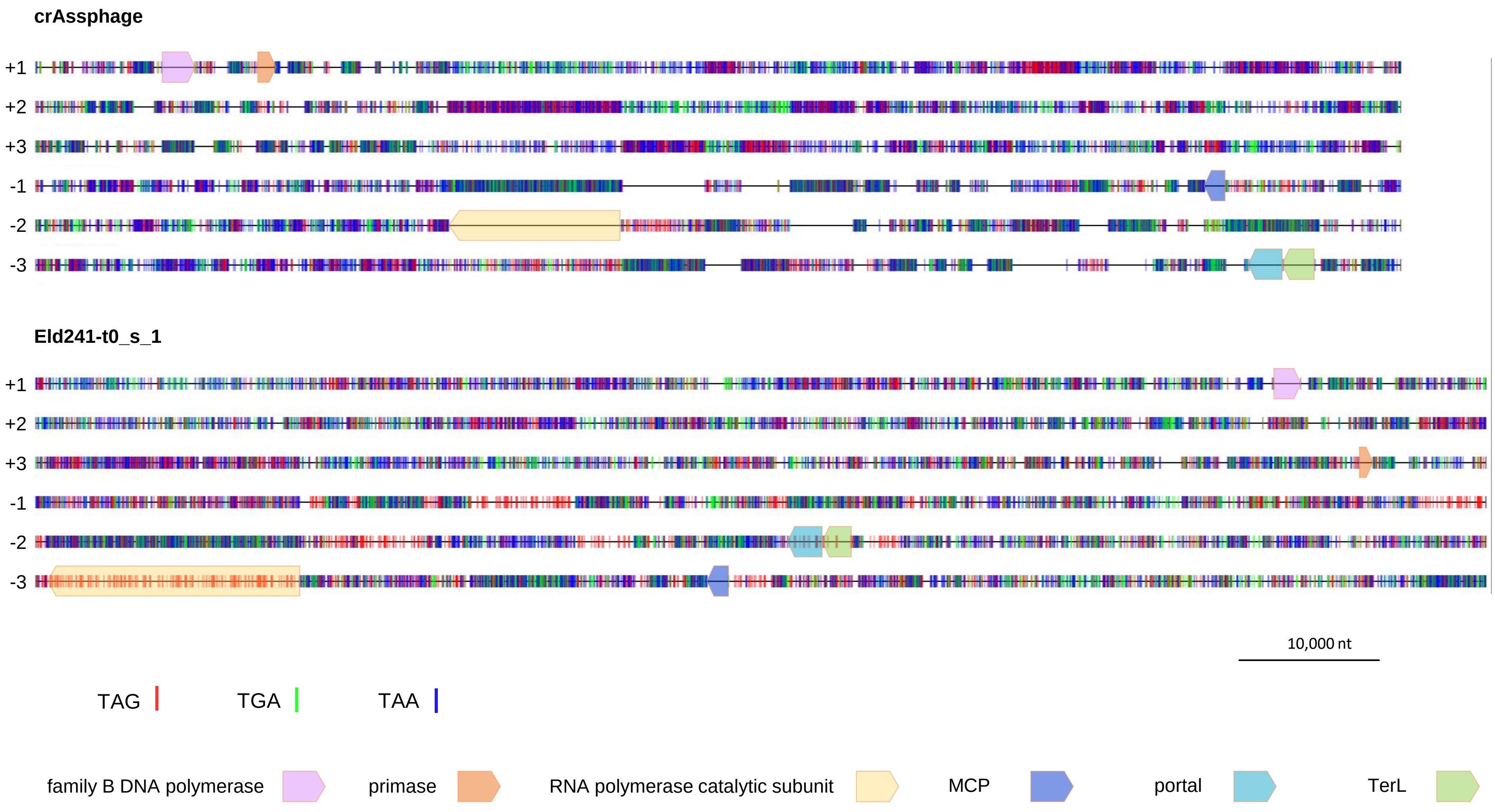

TAG, TGA, and TAA codons in the genomes of crAssphage and contig Eld241-t0_s_1 of Ref. 13

Supplementary Figure 8

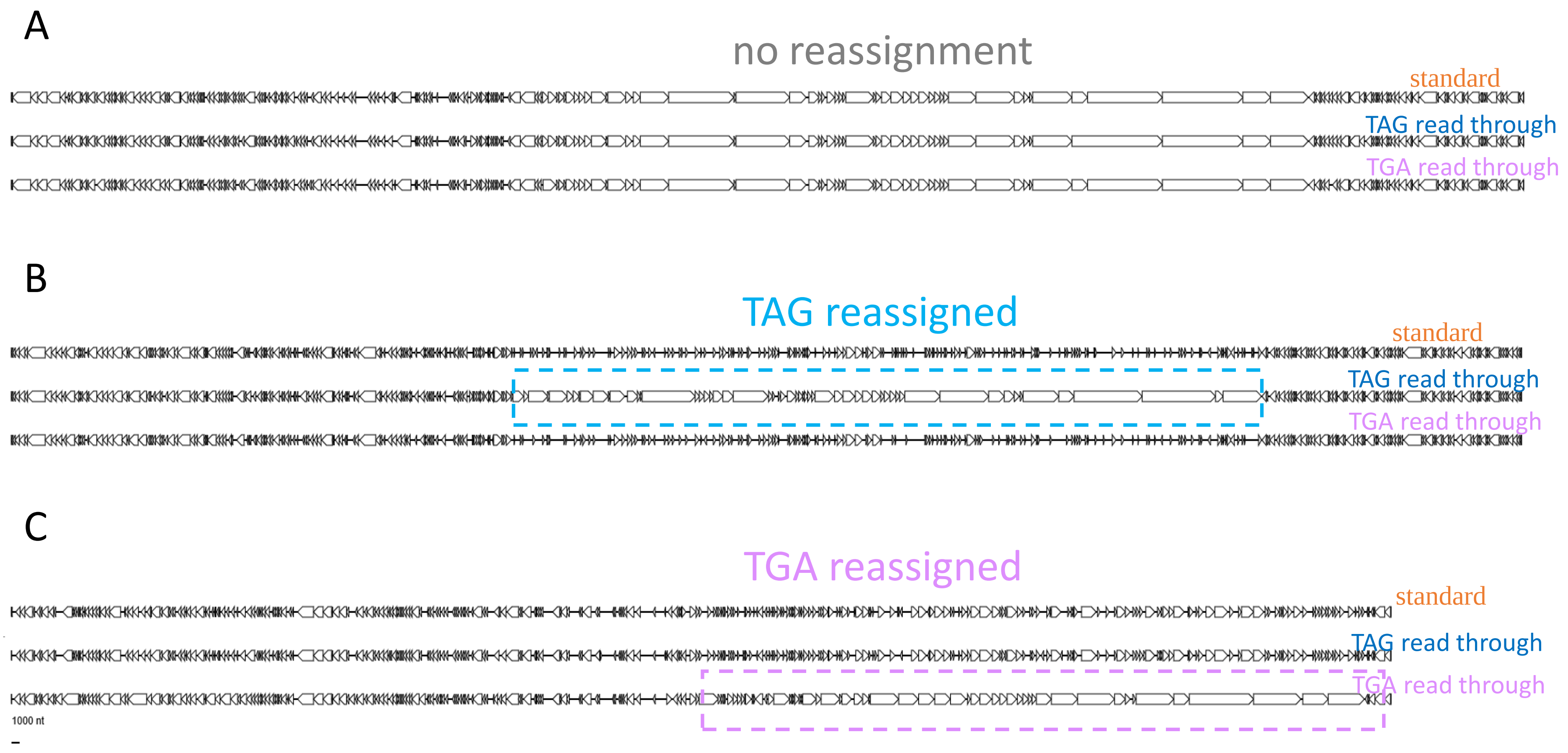

Three zeta group phage genomes, OLOE01000004 (A), OKSX01000010 (B), and OIXA01000019 (C), translated with standard bacterial code (top row), standard bacterial code with TAG read-trough (middle row), and standard bacterial code with TGA read-trough (bottom row). Accounting for read-trough TAG and TGA results in longer ORFs and higher coding density in parts of the OKSX01000010 and OIXA01000019 genomes.

Supplementary Figure 9

A

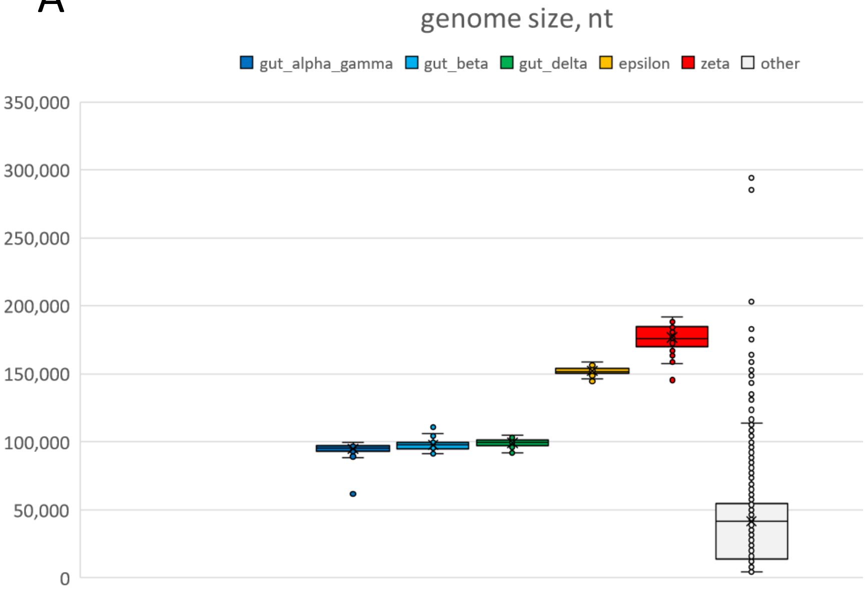

B

	# of contigs	# of tRNA	# of Sup tRNA	anticodon	reassigned codons
alpha gamma	190	115	1	TTA	TAA
beta	57	595	1	CTA	TAG
delta	233	472	153	CTA	TAG
epsilon	36	132	0		
zeta	79	1662	27	26 CTA, 1 TCA	26 TAG, 1 TCA
Flavob_phages	1	10	0		
other cMAGs	3343	2203	9	1 TCA, 2 CTA, 6 TTA	1 TCA, 2 CTA, 6 TTA

C

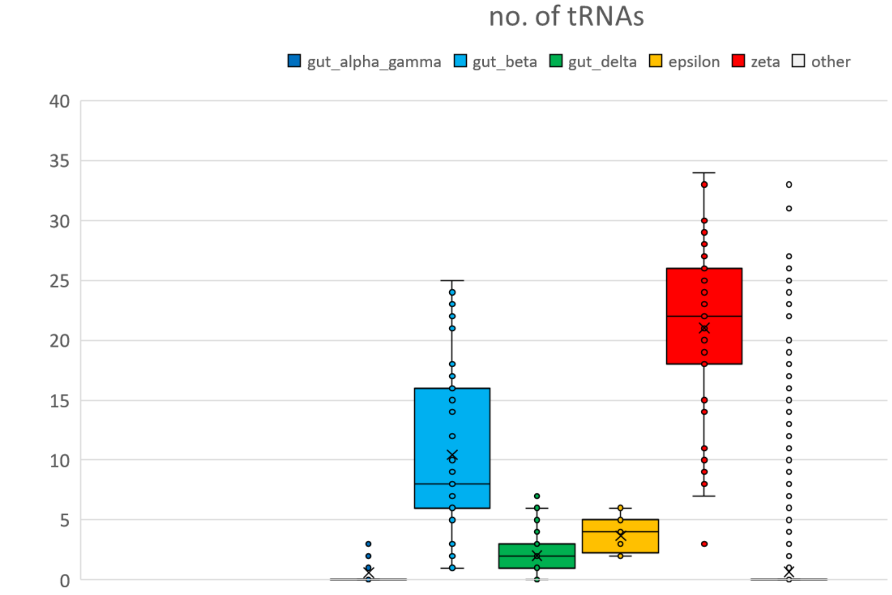

D

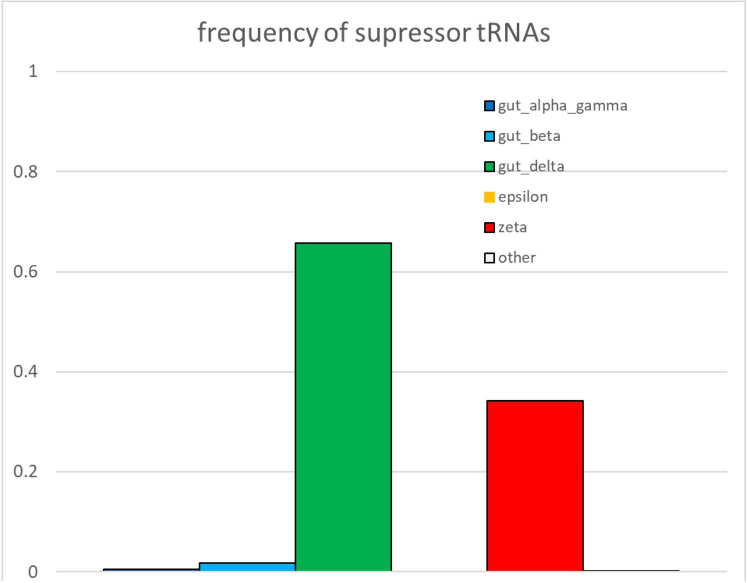

Supplementary figure 9

Genome size, number of tRNAs and suppressor tRNAs in crass-like cMAGs compared to other gut phage cMAGs (4-300 kb).

A. Distribution of genome sizes of alpha_gamma (n=190), beta (n=57), delta (n=233), epsilon (n=36), zeta (n=79) crass-like phages and other gut cMAGs (n=3344). Box boundaries correspond to the interquartile range (median indicated); whiskers indicate the lowest and the highest values within the range of 1.5 times the interquartile distance from the median; all points are shown.
B. Genome features of gut cMAGs.
C. Distribution of the number of tRNAs in the genomes of alpha_gamma (n=190), beta (n=57), delta (n=233), epsilon (n=36), zeta (n=79) crass-like phages and other gut cMAGs (n=3344). Box boundaries correspond to the interquartile range (median indicated); whiskers indicate the lowest and the highest values within the range of 1.5 times the interquartile distance from the median; all points are shown.
D. Frequency of suppressor tRNA occurrence in gut cMAGs.

Supplementary Figure 10

Emergence of suppressor tRNAs in crAss-like phages
A, TerL phylogenetic tree of alpha/gamma group phages.
B, a fragment of multiple sequence alignment of tRNA genes of 6 closely related genomes.

A

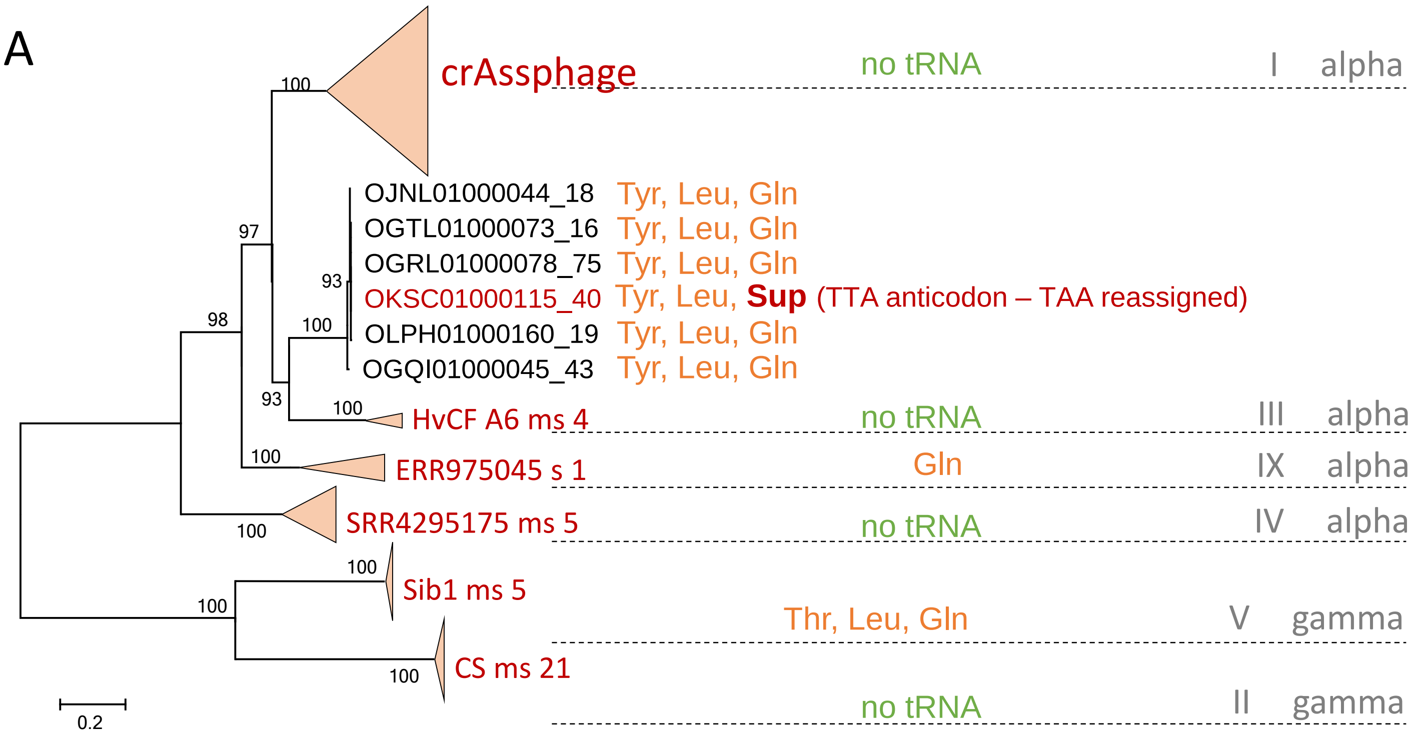

B

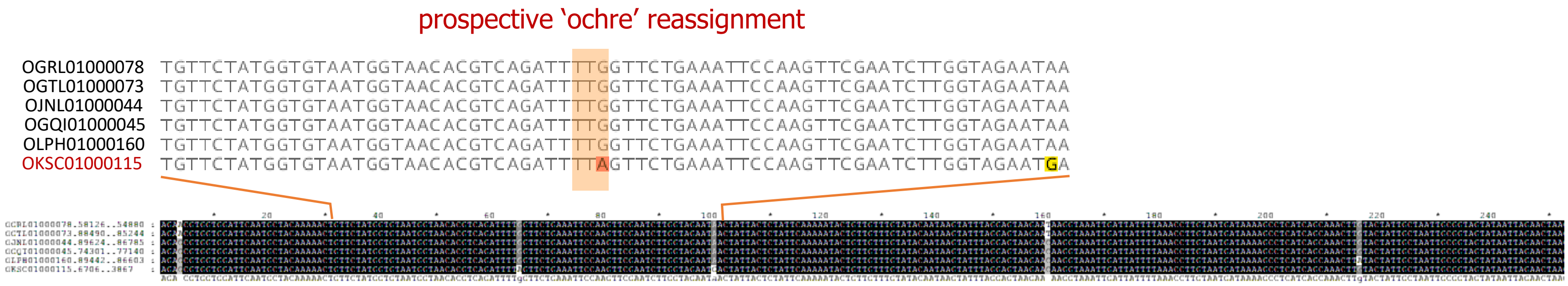

Supplementary Figure 11

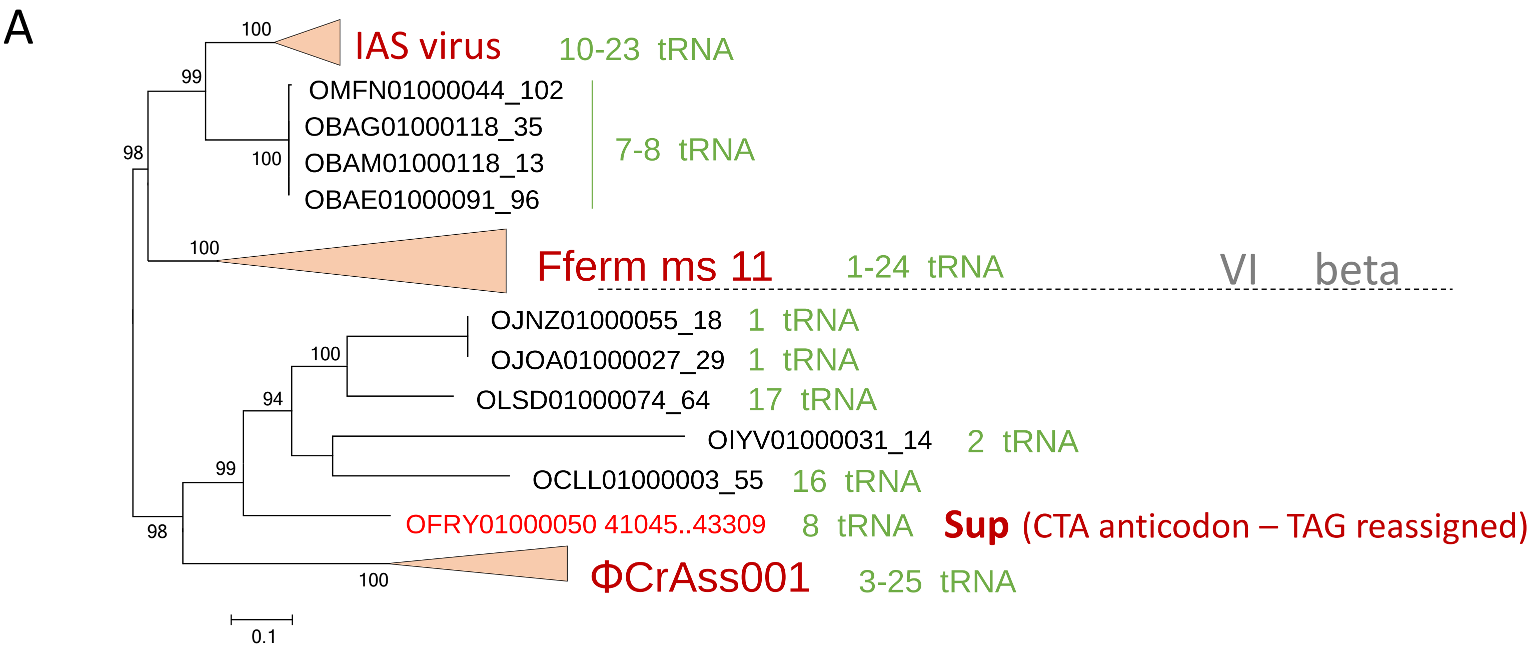

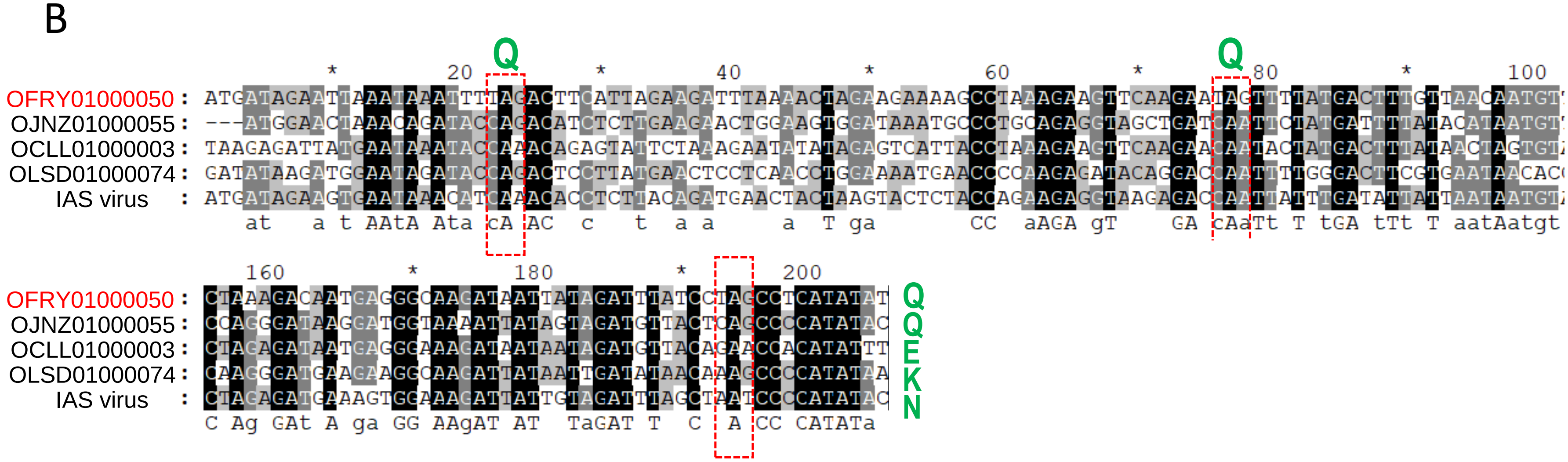

C

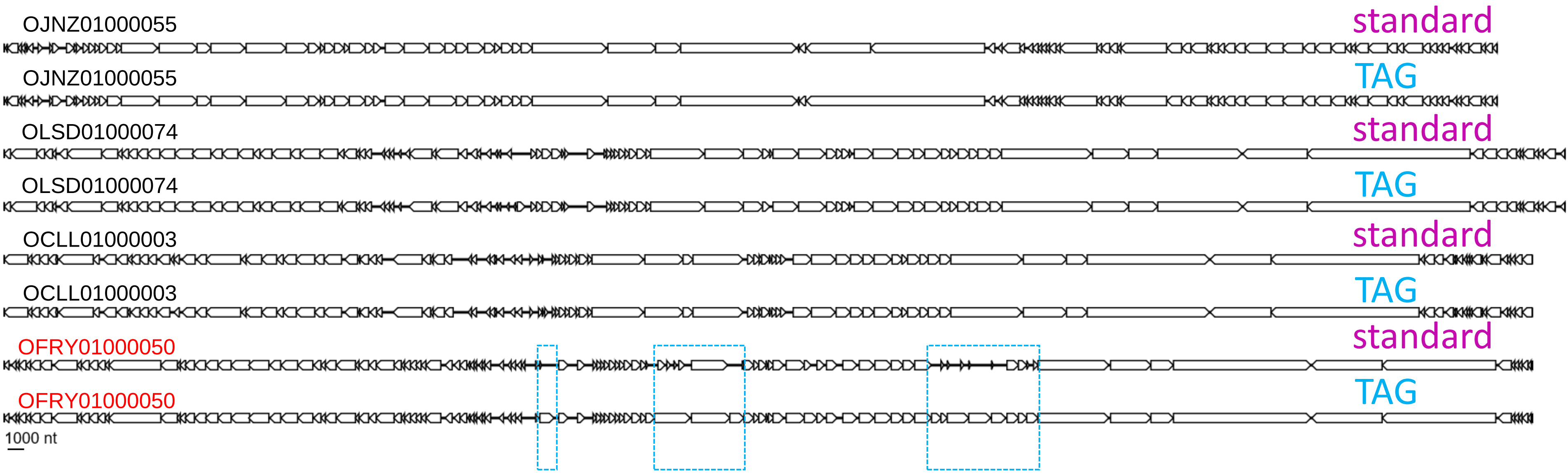

Suppressor tRNA in the Beta group of crAss-like phages.
A, TerL phylogenetic tree of Beta group phages.
B, A fragment of TerL gene alignment; the first three TAGs reassigned to Q in OFRY01000050 genome are shown; the whole TerL gene of OFRY01000050 has 15 reassigned TAG codons.
C, Emergence of alternative coding in OFRY01000050 genome. Four closely related genomes were translated with standard bacterial code and standard bacterial code with TAG read-trough. Regions of alternative coding in OFRY01000050 are marked with dashed boxes.

Supplementary Figure 12

Introns and inteins in crass-like phages. Whole genome maps for representative alpha/beta, delta, zeta, and epsilon phages are shown; whole-genome annotations of these genomes are here: ftp://ftp.ncbi.nih.gov/pub/yutinn/crassfamily_2020/

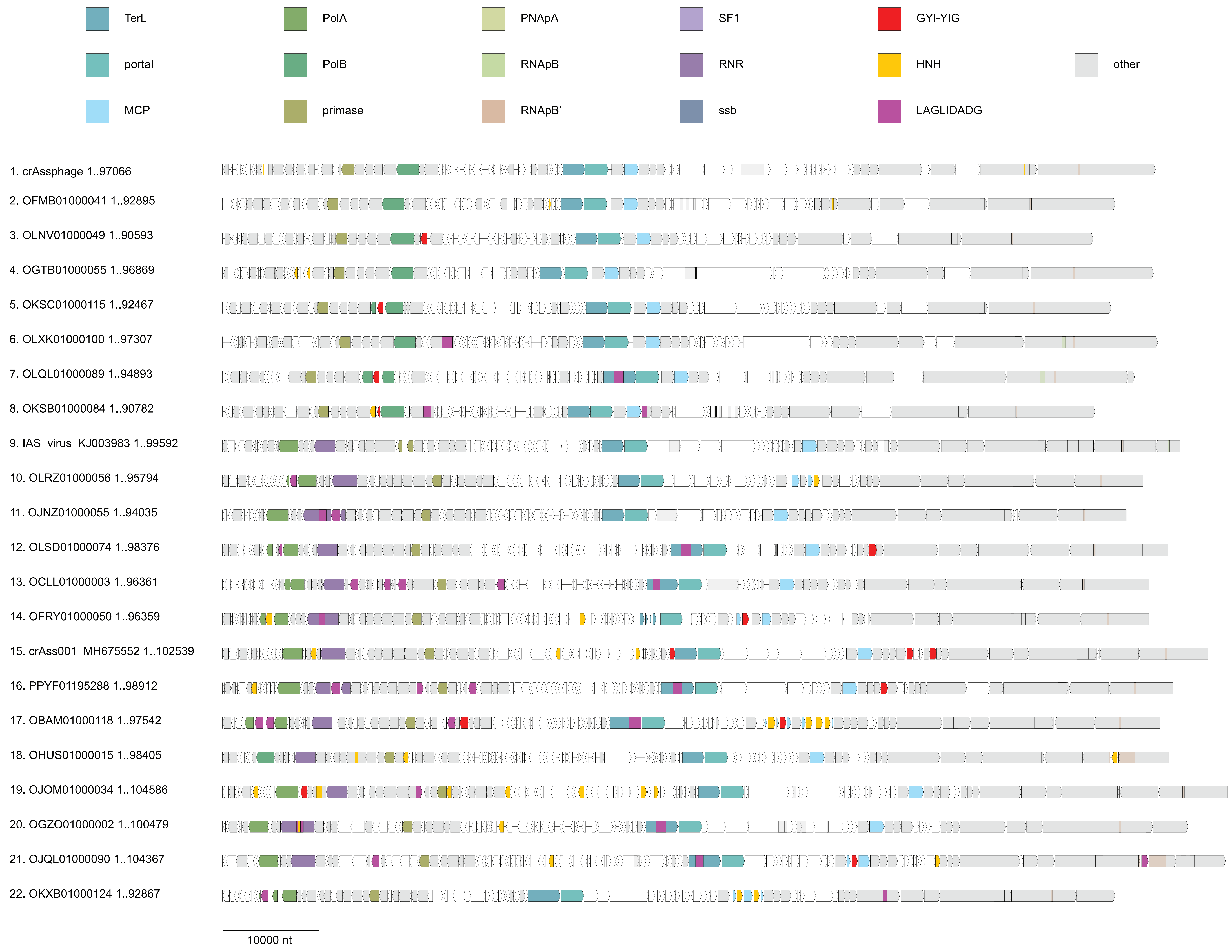

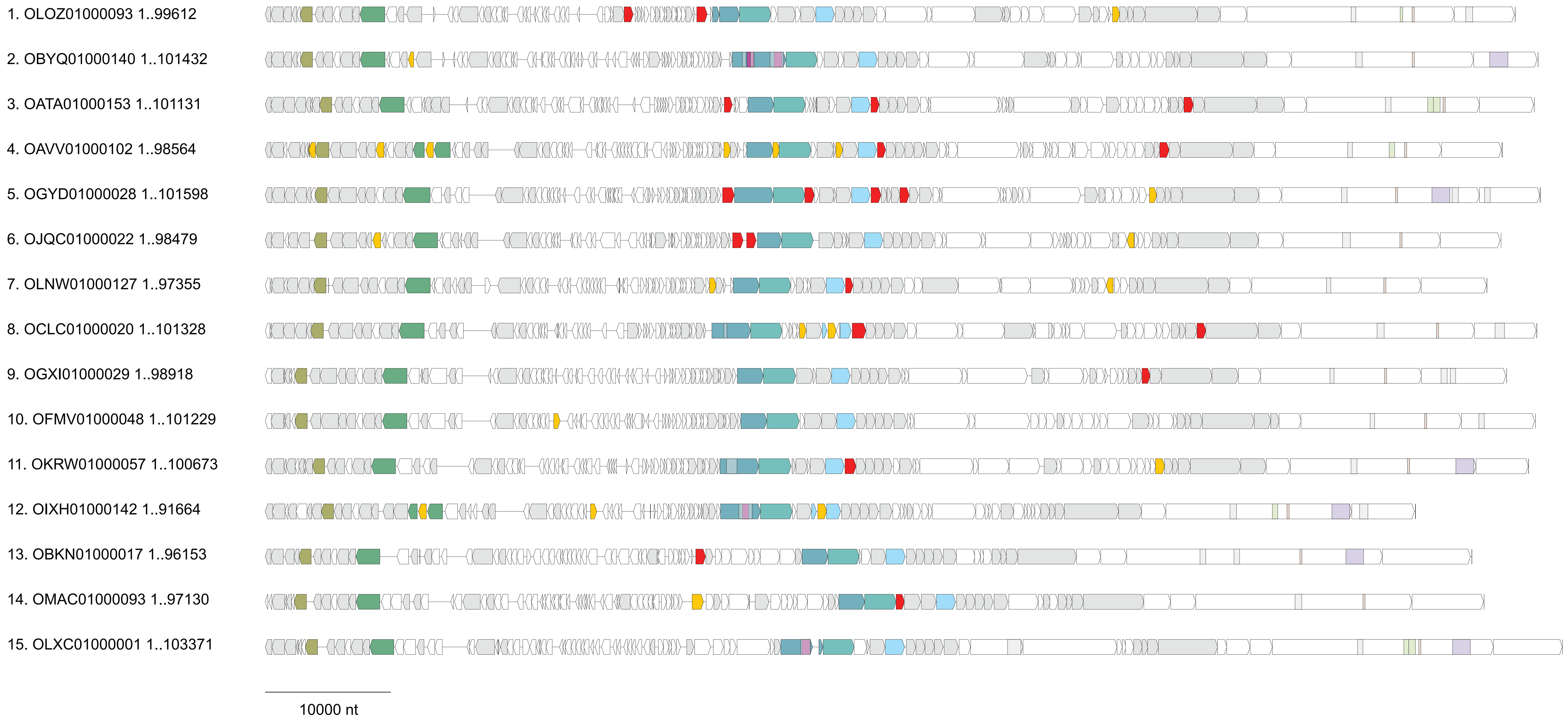
1. OLOZ01000093 1..99612
2. OBYQ01000140 1..101432
3. OATA01000153 1..101131
4. OAVV01000102 1..98564
5. OGYD01000028 1..101598
6. OJQC01000022 1..98479
7. OLNW01000127 1..97355
8. OCLC01000020 1..101328
9. OGXI01000029 1..98918
10. OFMV01000048 1..101229
11. OKRW01000057 1..100673
12. OIXH01000142 1..91664
13. OBKN01000017 1..96153
14. OMAC01000093 1..97130
15. OLXC01000001 1..103371
10000 nt

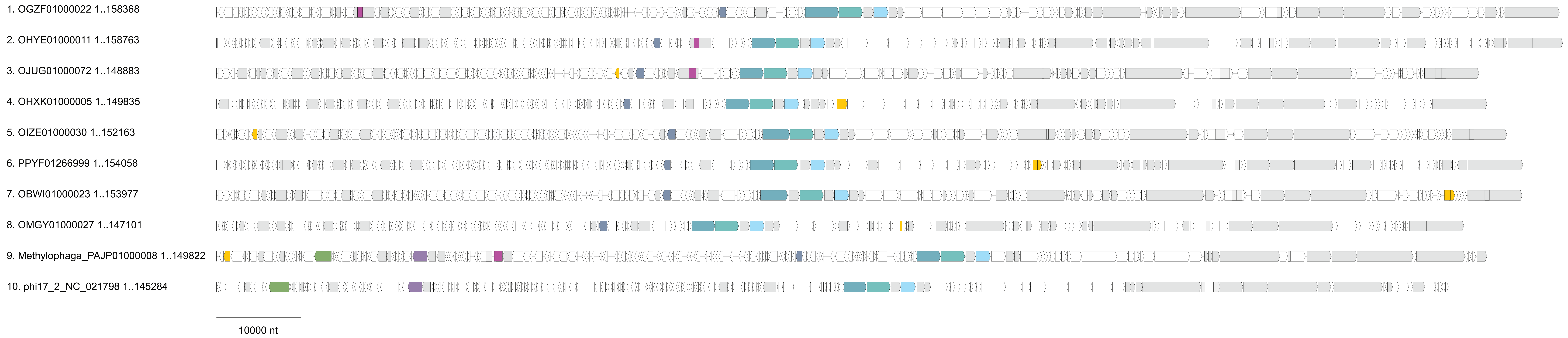

1. OGZF01000022 1..158368
2. OHYE01000011 1..158763
3. OJUG01000072 1..148883
4. OHXK01000005 1..149835
5. OIZE01000030 1..152163
6. PPYF01266999 1..154058
7. OBWI01000023 1..153977
8. OMGY01000027 1..147101
9. Methylophaga_PAJP01000008 1..149822
10. phi17_2_NC_021798 1..145284
10000 nt

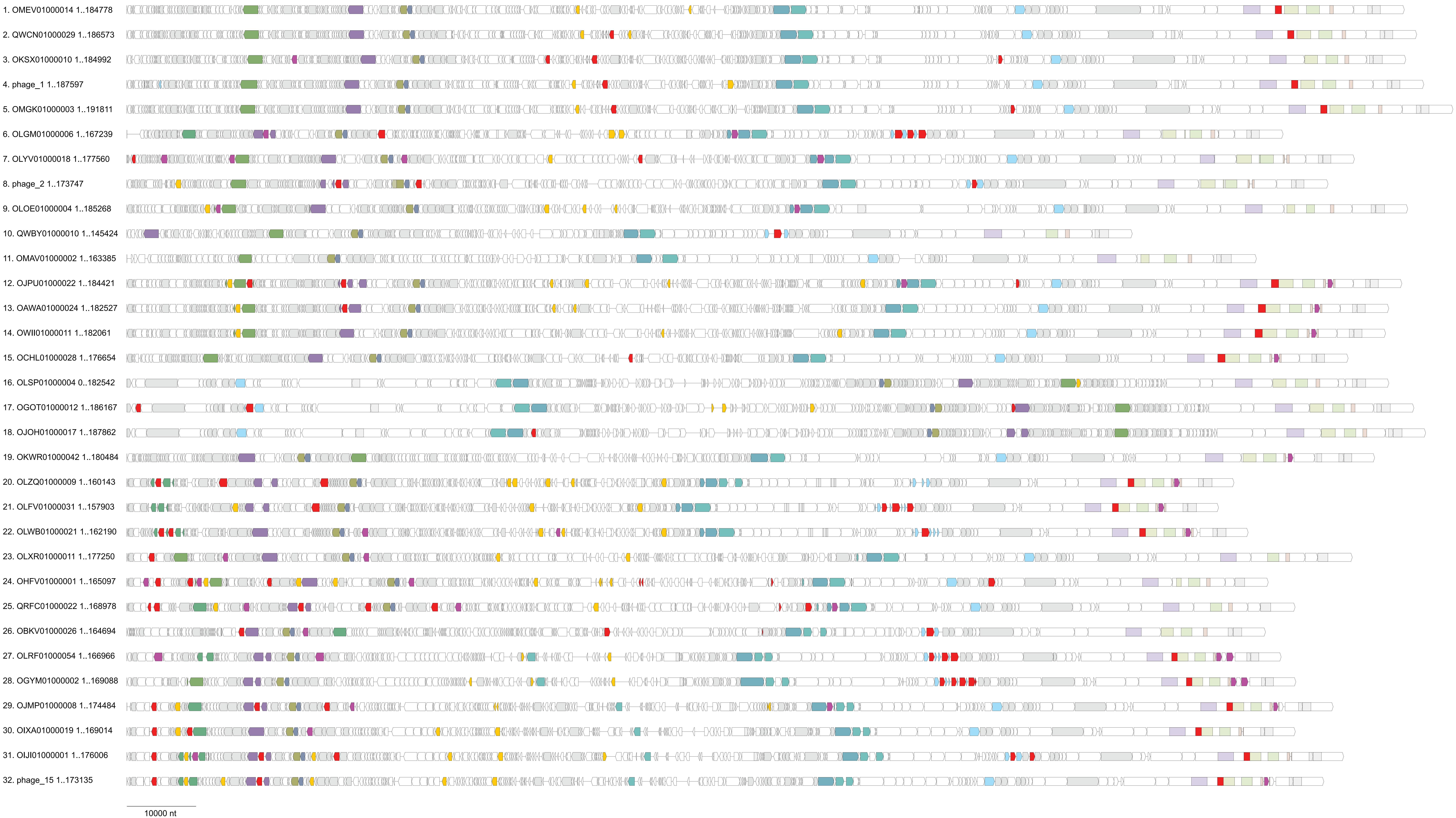
1. OMEV01000014 1..184778
2. QWCN01000029 1..186573
3. OKSX01000010 1..184992
4. phage_1 1..187597
5. OMGK01000003 1..191811
6. OLGM01000006 1..167239
7. OLYV01000018 1..177560
8. phage_2 1..173747
9. OLOE01000004 1..185268
10. QWBY01000010 1..145424
11. OMAV01000002 1..163385
12. OJPU01000022 1..184421
13. OAWA01000024 1..182527
14. OWII01000011 1..182061
15. OCHL01000028 1..176654
16. OLSP01000004 0..182542
17. OGOT01000012 1..186167
18. OJOH01000017 1..187862
19. OKWR01000042 1..180484
20. OLZQ01000009 1..160143
21. OLFV01000031 1..157903
22. OLWB01000021 1..162190
23. OLXR01000011 1..177250
24. OHFV01000001 1..165097
25. QRFC01000022 1..168978
26. OBKV01000026 1..164694
27. OLRF01000054 1..166966
28. OGYM01000002 1..169088
29. OJMP01000008 1..174484
30. OIXA01000019 1..169014
31. OIJI01000001 1..176006
32. phage_15 1..173135
10000 nt

Supplementary Figure 13

The transcription gene block in the Zeta group crAss-like phages. Conserved domains were identified with translating blast searches against a custom set of profiles and superimposed on genome map constructed with Prodigal.

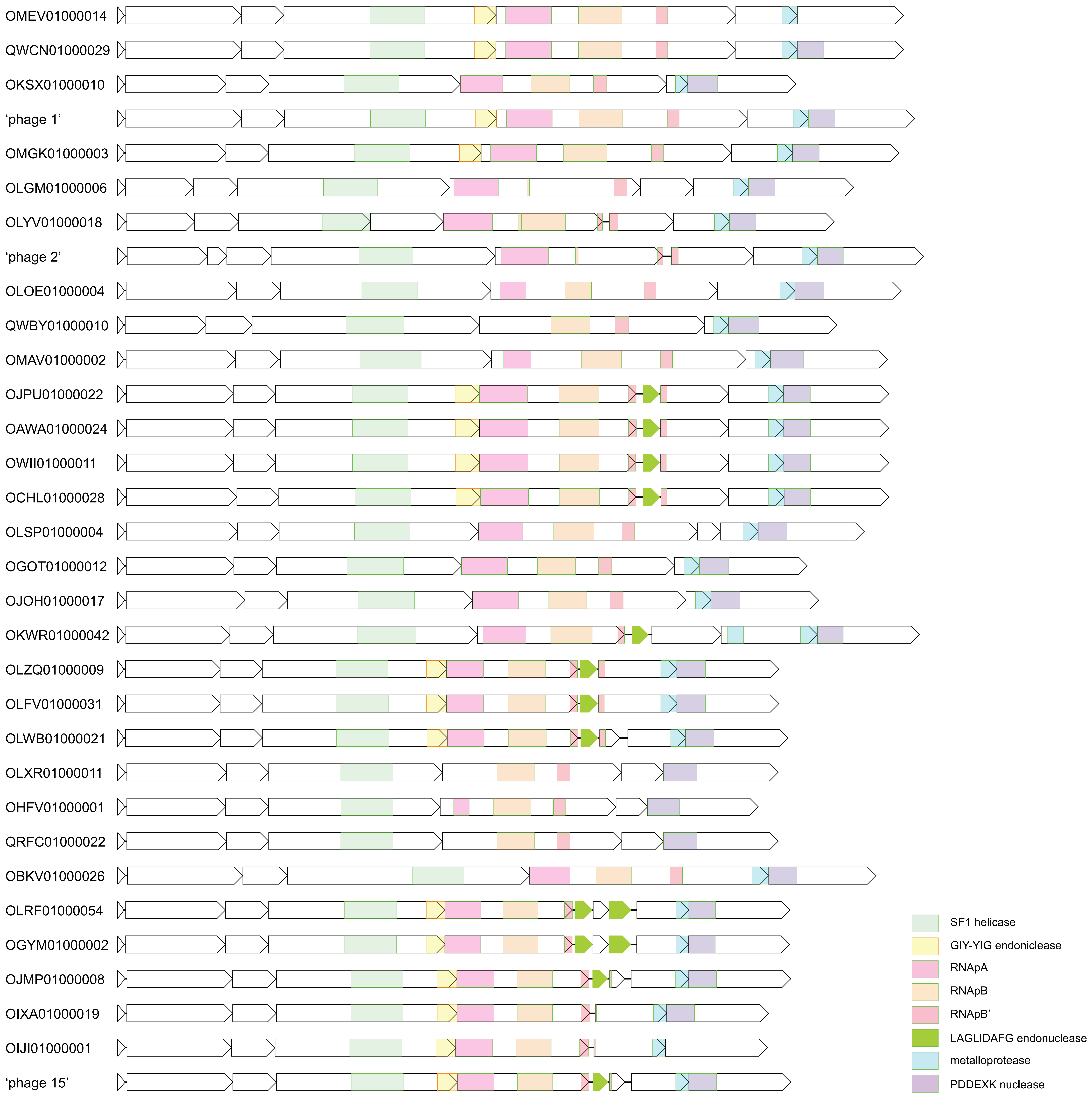

Supplement: Supplementary file 1 — Supplementary Information [file 41467_2021_21350_MOESM1_ESM.pdf]
