# Supplementary material for: Analysis of metagenome-assembled viral genomes from the human gut reveals diverse putative CrAss-like phages with unique genomic features
Source: Nat Commun. 2021 Feb 16;12:1044. doi: 10.1038/s41467-021-21350-w (PMC7886860; doi:10.1038/s41467-021-21350-w)
Supplement: Supplementary file 3 — Description of Additional Supplementary Files [file 41467_2021_21350_MOESM3_ESM.docx]

**Description of Additional Supplementary Files**
**Analysis of metagenome-assembled viral genomes from the human gut reveals diverse putative CrAss-like phages with unique genomic features**

Natalya Yutin^1^, Sean Benler^1^, Sergei A. Shmakov^1^, Yuri I. Wolf^1^, Igor Tolstoy^1^, Mike Rayko^2^, Dmitry Antipov^2^, Pavel A. Pevzner^3^, Eugene V. Koonin^1*^

^1^, National Center for Biotechnology Information, National Library of Medicine, Bethesda, Maryland 20894,USA;

^2^, Center for Algorithmic Biotechnology, Institute for Translational Biomedicine, St. Petersburg State University, 199004 St. Petersburg, Russia ;

^3^, Department of Computer Science and Engineering, University of California-San Diego, La Jolla, CA 92093, USA.

*Correspondence : [koonin@ncbi.nlm.nih.gov](mailto:koonin@ncbi.nlm.nih.gov)

File Name: Supplementary Data 1
Description: Annotation of the genome of crAss-like phages

File Name: Supplementary Data 2
Description: Abundances of crAss-like phages in human gut metagenomes
